# Surface Treatment of Polymer Membranes for Effective Biofouling Control

**DOI:** 10.3390/membranes13080736

**Published:** 2023-08-17

**Authors:** Vinita Vishwakarma, Jaya Kandasamy, Saravanamuthu Vigneswaran

**Affiliations:** 1 Centre for Nanoscience and Nanotechnology, Galgotias University, Greater Noida 203201, India; 2 School of Civil and Environmental Engineering, University of Technology, P.O. Box 123, Broadway, Sydney, NSW 2007, Australia; jaya.kandasamy@uts.edu.au; 3 Faculty of Sciences & Technology (RealTek), Norwegian University of Life Sciences, N-1432 Ås, Norway

**Keywords:** biofouling, polymer membrane, water filtration, nanomaterials, quantification

## Abstract

Membrane biofouling is the consequence of the deposition of microorganisms on polymer membrane surfaces. Polymeric membranes have garnered more attention for filtering and purifying water because of their ease of handling, low cost, effortless surface modification, and mechanical, chemical, and thermal properties. The sizes of the pores in the membranes enable micro- and nanofiltration, ultrafiltration, and reverse osmosis. Commonly used polymers for water filter membranes are polyvinyl chloride (PVA), polyvinylidene fluoride (PVDF), polyamide (PA), polyethylene glycol (PEG), polyethersulfone (PES), polyimide (PI), polyacrylonitrile (PAN), polyvinyl alcohol (PA), poly (methacrylic acid) (PMAA), polyaniline nanoparticles (PANI), poly (arylene ether ketone) (PAEK), polyvinylidene fluoride polysulfone (PSF), poly (ether imide) (PEI), etc. However, these polymer membranes are often susceptible to biofouling because of inorganic, organic, and microbial fouling, which deteriorates the membranes and minimizes their lives, and increases operating costs. Biofouling infection on polymer membranes is responsible for many chronic diseases in humans. This contamination cannot be eliminated by periodic pre- or post-treatment processes using biocides and other chemicals. For this reason, it is imperative to modify polymer membranes by surface treatments to enhance their efficiency and longevity. The main objective of this manuscript is to discuss application-oriented approaches to control biofouling on polymer membranes using various surface treatment methods, including nanomaterials and fouling characterizations utilizing advanced microscopy and spectroscopy techniques.

## 1. Introduction

Polymer membranes can be employed in a variety of ways for water treatment, purification, sea water desalination, chemical purification, etc. [1]. Many technologies are available to treat wastewater; however, membrane technologies are less expensive and easy to install, with fewer energy expenses. These membrane filtration methods are globally accepted and implemented to protect the environment and save energy, especially for desalination industries [2]. Membranes are used as separation techniques for various industries such as food, water, desalination, biogas plant, milk, and food production. Desalination is another process used to treat sea water or water containing salt by polymer membranes for various household, agricultural, or industrial applications. Biofouling is a serious problem in the case of its deposition on polymer membranes when used for water filtration units [3]. The undesirable growth of microorganisms and extracellular polymers on a membrane’s surface is problematic for water treatment or related industries. The aggressive attachment of fouling on a membrane’s surface leads to clogging, reduces filter efficiency, and damages the membrane [4]. This fouling mainly creates biomineralization and biomass growth on the membrane’s surface, which generates biocorrosion, scaling, microfouling, and macrofouling problems. Polymers serve as water filter membranes due to their good chemical, mechanical, and thermal properties [5]. These are synthetic polymers, namely, polyvinyl chloride (PVA), poly(acrylic acid) (PAA), polyvinylidene fluoride (PVDF), polyamide (PA), polyethylene glycol (PEG), polyethersulfone (PES), polyimide (PI), polyacrylonitrile (PAN), polyvinyl alcohol (PA), poly (methacrylic acid) (PMAA), polyaniline nanoparticles (PANI), poly (arylene ether ketone) (PAEK), polyvinylidene fluoride polysulfone (PSF), poly (ether imide) (PEI), etc. These polymers are classified based on their morphologies, structures, chemistries, and production procedures.

Polymer members are also separated on the basis of pore size, such as micro-, ultra-, nanofiltrate, and reverse osmosis [6]. A conventional polymer membrane has certain limitations in controlling biofouling problems. Materials that have been used to alter the polymer film and enhance its antimicrobial and chlorine resistance properties include polyethylene glycol, polyglycerol with PDA, chlorosulfonic acid, chloromethylation, sulfuric acid, polyamide, metal organic frameworks (MOFs), Zwitterionic, Polydopamine, and nanoparticles such as SiO_2_, TiO_2_, and ZnO (Table 1). One study showed that multiwalled carbon nanotubes were added to polyether sulfone due to their tubular structures and high surface areas to control fouling attachments on a membrane’s surface, and gum arabic was added as a natural surfactant to enhance the antimicrobial properties. Membranes synthesized with carbon nanotubes enhanced their antifouling performance along with their mechanical strengths and thermal stabilities because of their enhanced surface area, hydrophilicity, and porosity [7].

**Figure 1:**
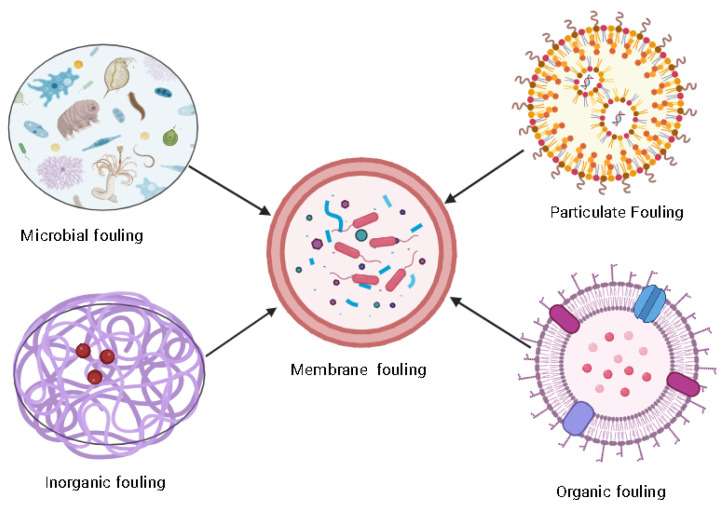
Types of polymer membranes fouling.

Nevertheless, these polymers always encounter foreign element depositions on their surfaces. Overall, microbial fouling initially forms a biofilm layer on the polymer’s membrane and forms a colony-like structure by secreting extracellular polymeric substances (EPS). EPS contains large amounts of molecular weight organic compounds comprising proteins, lipids, nucleic acids, and polysaccharides [8]. This membrane should be resistant to pH, temperature, ionic charges, hydrophobicity, membrane pore size, water flow rate, antimicrobial activity, etc. Principally, polymer membranes are loaded with four different types of fouling due to feed water (Figure 1):

(i) Inorganic fouling is due to the precipitation and deposition of minerals, salts, oxide, hydroxide, etc.;
(ii) Organic fouling includes proteins, polysaccharides, nucleic acids, fatty acids, etc.;
(iii) Particulate fouling is due to the deposition of solid particles;
(iv) Microbial fouling consists of aggregates of microbes deposited on the membrane’s surface.

Microorganisms multiply, grow, accumulate inside biofilm structures and develop complex structures [9]. The uncontrolled growth of biofilms promotes anaerobic bacteria inside their structures, which is the reason why corrosion occurs on metal surfaces. It spreads like a mat, which has an adverse effect on the membrane system due to its mechanical damage and the production of poor-quality clean water [10]. Biofouling has many side effects on human health because it is persistent and causes chronic diseases. Dental plague formation, dental caries, implant infections and deterioration are common problems due to biofouling contamination. Almost all industries, such as the food, medical, pharmaceutical, bottling and wine, textile, construction and water sectors, are significantly affected by biofouling formation in their components. Routine preventive measures, for instance mechanical and chemical treatment to avoid biofouling and scaling and chemical deposition on membranes, are not very successful in enhancing the durability of polymer membranes. Routine biofouling treatments are generally performed by disinfectants using chlorine, ozone treatment, silver, hydrogen peroxide, copper sulphate, chloramines, ultraviolet light, photocatalyst materials, disinfectant dose in the form of continuous versus shocking dose, concentration of organic and inorganic compounds, chlorine dioxide as biocide, etc. [11]. Non-oxidizing biocides are also employed for microbial treatment.

Chlorination doses from 0.2 to 1 mg/litre are effective in disinfecting bacterial contamination; however, doses greater than this limit pose a carcinogenic threat. Ozone is used for water disinfection to avoid the contamination of protozoan cysts. Silver has the ability to degrade water pollutants since it is known for its antibacterial properties, but it is costly and more processing time is needed to treat water. Ultraviolet light treatment physically degrades microorganisms by destroying their nucleic acids and reduces bacterial growth. UV treatment struggles to achieve an optimal dose but there is no detrimental product or outcome. Pulsed laser treatment is also effective in killing microorganisms by applying an accurate amount of energy through voltage electricity. Photocatalyst materials such as TiO_2_ and ZnO degrade inorganic and organic contaminants from water, such as cyanides, nitrates, nitrites, and humic acid. Natural amino acids such as lysine were used as coating agents on the filtration membrane to avoid bacterial attachment [12]. Surface treatment is a common phenomenon that improves the surface of any material, especially when this is a major issue in biofouling problems. Titanium (Ti) metals, which are used as condenser tube materials in nuclear power plants, have been modified by pulsed laser deposition techniques with Cu and Ni nano thin films. This has indicated an apparent decline in bacterial attachment on Ti surfaces [13]. Carbon steel (CS), as a pipeline material, has more difficulty avoiding microbial invasion; however, Cu-Ni-Zn coatings were developed on its surface for the effective reduction in sulfate-reducing bacterial attachment [14]. Likewise, the surface treatment of polymer membranes to avoid the antiadhesion of microorganisms is needed. This manuscript is a systematic study of the surface treatment of polymer membranes describing the various methods used to select the relevant materials as well as their mechanism, including nanomaterials.

**Table 1:** Materials used for membrane modification.

Materials for Membrane Modification	Advantages
Membrane modified with polyethylene glycol	Arrest adsorption process [15], hydrophobicity [16]
Polyglycerol with polydopamine (PDA) coatings	Antifouling and resistance to bacterial adhesion [17]
PSF then poly(arylene ether ketone) membranes are altered with chlorosulfonic acid, chloromethylation, sulfuric acid, etc.	Attachment of hydrophilic group, anticoagulant antibacterial [18,19]
Poly(4-vinylpyridine-coethylene glycol diacrylate) deposition on RO membrane	Reduced bacterial attachment [20]
Thin film composite polyamide membrane improved with amine terminated sulfonated poly(arylene ether sulfone).	Hydrophilic group on membrane surface [21]
Metal organic framework (MOFs)	Heat resistance, high surface area, permeable with enhanced flow rate [22]
Zwitterionic chemical based modification	Fouling control [23]
Polydopamine coating on polypropylene membrane	Reduce the waster contact angle by 110° to 67° and improve hydrophilicity of membrane [24]
Inorganic nanoparticles such as SiO_2_, TiO_2_, ZnO reinforced in polyvinyl chloride, polyvinyl alcohol	Enhanced performance of membrane and its antibacterial activities [25]

## 2. Surface Treatment of the Polymer Membrane

Drinking water always has a risk of contamination caused by microorganisms, and 11% of the world’s population does not have good water to drink. Contaminated water filtration through polymer membranes is significant and easy to clean with lower costs. Traditionally, the protection of membrane filtration from organic loads and biofouling follows pre-treatment processes such as adsorption, oxidation, ion exchange, and membrane filtration [26]. This pre-treatment process is essential to remove the contaminants from membrane filtration. This process improves the efficacy of the membrane and the productivity of good-quality water [27]. Polymer membranes have a tendency to damage their surfaces because of their structures, materials and chemicals, as well as differences in charges and contact angles. To impart the specific properties to polymer membranes, the surface treatment of membranes by various chemical modifiers with precise techniques is essential to minimize biofouling attachment, reduce the water contact angle and maintain hydrophilicity.

An antimicrobial polymer membrane was prepared with the grafting method by inducing silver nanoparticles on a sulfonated membrane to enhance the hydrophilicity, which activated the membrane surface [28]. The grafting of polymers by hydrophilic methods avoids biofilm formation on the membrane surface [29]. The fabrication of a composite electrospun ultrafiltration membrane of PVA-PAA on top of polysulfone (PSU) has curtailed organic fouling and increased hydrophilicity and other functionalities [30]. Metal–organic frameworks (MOFs) incorporated with thin film nanocomposite membranes have potential applications in molecular separation [31] (Table 2). MOFs are innovative hybrid materials consisting of clusters of metal ions and organic linkers [32]. Due to their precisely defined porous structures and intriguing properties, MOFs have emerged as promising nanofillers for membrane applications [33].

Polymer membrane surface treatment has also been reported through plasma ionized gas, which induced atomic excitation in polymer atoms, with the ultimate aim of creating a fouling-resistant polymer surface membrane [39]. The blending of basic polymers with various inorganic nanoparticles has shown better surface chemistry on the membrane [40]. The blending of TiO_2_, Ag, graphene oxide, mesoporous silica, alumina, Zr, Cu with PES, PsU, PVDF, PAN, etc., improved the mechanical strength, hydrophilicity, permeability, porosity and antifouling properties. These nanoparticles have better characteristic properties than bulk materials because of their enhanced surface-to-volume ratios. TiO_2_ nanoparticles have superior self-cleaning, photocatalytic, hydrophilicity, thermal and chemical properties, which will be helpful for polymers to avoid attachment. However, even though there is a chance that agglomeration will occur, the exposure of nanoparticles to the environment can be prevented. Ag nanoparticles are less toxic and kill the bacteria by blocking their respiratory enzymes and preventing their attachment on the polymer membrane. These nanoparticles were blended with PES by immersion precipitation techniques and an antifouled membrane was achieved [41]. Graphene oxide fabricated with polymer membranes limits water contamination [42]. Nontoxic coatings using silicone protect the surface from the attachment of large microorganisms; however, because of its weak mechanical strength, this coating is not stable. The surface of the polymer membrane treated with proteolytic enzymes degrades the extracellular polymeric enzymes. One study reported that natural lysozyme enzymes break down the peptidoglycan of the bacterial cell wall [43]. Surface treatment techniques of different types of polymer membranes and their applications are summarized in Table 3.

## 3. Base and Consequences of Membrane Biofouling

Biofouling has an impact on almost all environments and industries. Water industries, oil and gas pipelines, bioimplants, food, bottling, concrete, paper and pulp, power plants, and dairy industries experience regular biofouling in their components. The sequence of fouling on the membrane surface commences with the wet and moist surface, where it creates the necessary environment to form a biofilm. Then, the process of the transfer of microbes begins on the film and firmly attaches to it through metabolic activities. Fouling develops on the membrane due to physicochemical exchanges that involve water and materials of the polymer membrane [14,47,48]. The growth of fouling occurs on membrane surfaces as well as the inner surface. The main reasons for membrane biofouling and its degradation are pH, temperature variations, scale formation, suspended solids and the oxidation process of water chemicals such as chorine, hydrogen peroxide, other chemicals and the growth of microorganisms. The difference in temperature from 35–45 degrees Celsius is another reason for the thermal damage to the membrane.

The community analysis of biofilms is significant for identifying the presence of microorganisms at the genus and species levels. Microbes such as bacteria and fungi and the presence of suspended solids such as clay, iron, silica, manganese and aluminium block water flow in membrane systems. There are various types of foulants such as flocs, microorganisms, and scales. The routine cleaning of mechanical and chemical methods is implemented by back-flushing the membrane and mild chemicals such as detergents, acids, and anti-sealants. However, these methods are temporary solutions to control fouling attachment on membrane systems. Nevertheless, a high concentration of feed water increases the pressure on the membrane, which leads to high electricity consumption. Biofouling causes mechanical damage to the membrane due to variations in water pressure, formation of air bubbles, turbulence in water flow, shaking-like pores and cracks. Currently, the real-time prediction of biofouling on polymer membranes using artificial intelligence is receiving great attention.

## 4. Quantification of Polymer Membrane Biofouling

The physical and chemical properties of polymer membranes have been characterized through advanced techniques [49]. This is achieved using imaging techniques to confirm its structures and the other parameters required for its characteristics. These techniques are suitable for interpreting how well membranes function. Microscopic and spectroscopic techniques are involved in the direct and real-time analysis of membrane biofouling. These techniques provide detailed information about biofilm deposition as well as its association with the polymer membrane. Detailed information on biofilm deposition on polymer membranes is required to understand their performance, which is modified by surface treatment. Some of the characterization tools are described in more detail below.

### 4.1. Epifluorescence Microscopy

Epifluorescence microscopy is a fast and simple tool for the quantification of biofilms. It provides exhaustive information about the origin and morphology of biofouling and the structural characteristics of the specimens. Metal specimens were prepared using 0.1% of the fluorescence stain acridine orange, which emits green fluorescence when intercalated with DNA upon excitation at 480–490 nm, and orange–red fluorescence is obtained when acridine orange complexes with RNA [6]. The total viable count on the membrane filter enumerates the microcolony [50]. Hence, epifluorescence microscopy is a promising approach for observing and quantifying biofilms on polymer membranes [51].

### 4.2. Scanning Electron Microscopy (SEM)

The basic principle of SEM is similar to that of optical microscopy; however, electrons are used as detectors to investigate the SEM image, and they are also compared with the magnification difference. SEM is the technique in which biofilm specimens are analysed when grown in solid substrate. It captures the images with good spatial determination. SEM provides useful information on the structure of contaminated polymer membranes as well as the steps of the biofilm development process [52]. SEM analysis is undertaken for the conductive specimens coated with gold. SEM also analyses the membrane’s porosity and permeability [53]. This instrument analyzes the information during the process of cleaning the membrane [54].

### 4.3. Transmission Electron Microscopy (TEM)

TEM is a tool that observes the membrane surface at higher magnification in nanometers, where the transmitted electrons convert into an image. The sample preparation of polymer membranes for TEM imaging is difficult because they are soft materials. Generally, soft membranes are immersed in liquid nitrogen to harden them before imaging [55]. Wet and soft samples evaporate during TEM analysis due to the high vacuum.

### 4.4. Atomic Force Microscope (AFM)

AFM is emerging as a very potent alternative tool to study the presence of fouling on membrane surfaces. It provides roughness, porosity and 3D images of the sample surface [56]. Here, the specimens are scanned with a cantilever attached by a silicon tip, and the image is plotted. Compared to SEM and TEM, AFM has a lower depth of field but better image information about the surface topography of bacterial cells. Other imaging techniques such as electron microscopy, fluorescence-labelled CSLM, magnetic resonance, and scanning transmission X-ray microscopy are used to visualize biofilms. However, AFM produces information on a nanoscale basis, enabling us to understand bacteria–mineral interactions [56]. AFM provides 3D images of surface topography and quantitatively measures the interactions and cohesion of biofilms in the form of qualitative images [57].

### 4.5. Surface Enhanced Raman Spectroscopy (SERS)

SERS is an exceptional technique used to identify the formation of dual species and characterize dynamic transformation in dominant species of biofilm [58]. It provides in-depth information about the biofilm composition, its development and the presence of biomolecules in biofilms [59]. SERS identified biofoulings as a mass on spiral-wound reverse osmosis membranes [60]. A study confirmed that Raman spectroscopy analysed the presence of nucleic acids, proteins, and carbohydrate EPS in biofilm colonies formed by Pseudomonas species [61,62].

### 4.6. Confocal Laser Scanning Microscopy (CLSM)

CSLM is an important technique for studying biofilms at the accumulation stage, particularly biofilm matrices, since it makes the real-time imaging of entirely hydrated, living specimens possible. This is a commonly used technique to detect the emission of fluorescence as 3D structures of biofilms [63]. Biofilm structural properties were studied by quantifying the biofilm, its thickness, volume and roughness [64,65,66]. This instrument also elaborates on the consequence of chemicals present on the biofilm, which helps ti develop the protocols for cleaning biofilm materials [67].

### 4.7. Fourier Transform Infrared Spectroscopy (FTIR)

FTIR is a non-destructive practice to identify the functional groups of organic compounds present in membrane fouling [68]. Microscopic techniques are unable to capture the details of biofilm composition, whereas FTIR detects the fast formation of biofilms in membrane systems. Generally, biofilm quantification is performed by serial dilution through colony-forming units, but accurate qualitative analysis is executed by FIIR. FTIR also analyses the reason for membrane degradation, while its structural arrangement recognizes adsorbed foulants and their effects on the membrane [69]. FTIR generates information on the molecular and chemical composition of biofouling [70].

### 4.8. Nuclear Magnetic Resonance (NMR) and Magnetic Resonance Imaging (MRI)

NMR was used to examine the structural details of the biofilm grown on the substances; however, this technique is not suitable to obtain information about the high molecular weight and complex nature of biofilm [71]. Both NMR and MRI instruments are non-invasive and are used as 3D imaging tools to investigate membrane biofouling geometries [72].

### 4.9. Thermography

Thermography is a highly versatile, non-destructive and low-cost infrared camera imaging technique that maps the biodeterioration of materials similar to polymers, concrete, different types of metals, stones, rock, pipelines, etc., without any specific temperature. This method has been used to obtain infrared images from the entire membrane fouled surface and its succession because of continuous thermal excitation [63]. The fouled membrane was studied in the excitation mode of thermography, and images were captured by IR radiation [73] in the form of a temperature assessment. Compared to SEM and AFM, this technology is still nascent but nonetheless has the potential for biofilm characterization, as well as being easy to use and less expensive.

## 5. Conclusions and Future Directions

Polymer membranes have received great attention for water purification because no chemical or energy is needed. Fouling is a serious issue in the use of membrane technology for industries. It needs to be properly addressed during the fabrication of the water filtration unit for operation so that no difficulties ensue. Biofouling growth is a common problem in unwanted places and is harmful to human beings and industrial components. Fouling of the membrane is a complex event and proper action has not yet been taken to solve it. The prevention of bacterial attachment to the surface at the initial stages is important and requires the treatment of primary feed water, which will reduce the bacterial contamination and its feed content. The formation of biofouling is a major issue in polymer membrane systems, which reduces their durability, permeability and ultimately lifetime. This has an effect on the elevated operating process in terms of pressure and frequently routine chemical cleaning rate. Overall, fouling reduces the quality of clean water, disrupts the membrane system process and has an effect on its cost. Clean water is a global concern, as billions of people need safe and fresh water for food, agriculture and electricity generation.

Long-term conventional cleaning methods are followed to eradicate biofilm from the roots of the surface of the substrate using either physical, chemical or mechanical methods to control, to a certain extent, biofilm formation. The chlorination process used to avoid microbes on the polymer surface is not very effective and, in due course, this process weakens the polymer network. Other techniques, such as ozone treatment, UV light, and photocatalysis, have their own limitations in controlling biofouling. To maintain consistent water quality, the implementation of membrane technology is important so that the contamination caused by microorganisms is avoided. Both academic and industrial sectors have focused more research on membrane technology, and the market for its growth is expanding. A systematic plan is required to monitor, detect and control the growth of biofouling based on the environment and composition of biofouling.

Polymeric membrane contamination due to biofouling has not been researched properly. This paper critically reviewed and focused on different methods of the surface treatment of polymer membranes to avoid various types of contamination, especially fouling attachment on the membrane surface. The protection of polymer membranes through biofouling contamination is achieved by selecting suitable membrane materials, pre-treatment, optimizing the operating procedure, and periodic cleaning, which will help to maintain the membrane for a long time. All these parameters need to be maintained to safeguard the environment, reduce the consumption of the natural environment and avoid negative health effects. The main strategies to be followed to prevent fouling on the surface of polymer membranes require different treatment techniques.

The fouling process in any environment and surface is a repeated process if it is not treated properly. Therefore, an advanced approach is important to modify the surface of polymers. The surface treatment of polymers is a common process to obtain the desired efficient properties. Many tools and techniques are available to understand the fouling properties on membrane surfaces and their treatment methods. An evaluation of biofilm and its characterization can be achieved through relevant microscopy and spectroscopy. Coating, blending, and grafting represent some desirable approaches to prevent polymer membranes from biofouling. Although several developments and techniques are available for the surface treatment of polymer membranes and their protection from biofouling growth, many issues need to be addressed in the future for the instant detection of biofouling. The development of sensor devices to detect moisture, pH, temperature, etc., or by observing biofilm metabolites, will constitute a suitable strategy for the early detection of fouling. Nevertheless, the implementation of innovative techniques such as artificial intelligence systems are required to predict fouling formation on the surface of polymer membranes. Future research must focus on minimizing the polymer membrane surface roughness and charge for the efficient control of biofouling. In the future, better-modified polymer membranes with novel materials must be developed for long-term duration based on societal, environmental and economic needs.

## Figures and Tables

**Table 2:** Summary of recent studies on MOF-incorporated TFN membranes for liquid separation [31].

Incorporated MOFs	Pore Size	Particle Size	Membrane Used	Pressure Applied (bar)	Pure Water Productivity (L/m^2^h/bar)	Rejection for the Solution 2 g/L of NaCl	Ref.
(Cu-THQ) MOFs	1.1 nm	30–70 nm	RO OSN	15.0 4.0	1.2–2.9 12.2–16.9	98.8–98.9%	[34]
DMF Allura Red Ni-MOFs	<0.4 nm	N.A.	RO	20.0	1.03–2.50	99.3–99.2%	[35]
ZnTCPP	N.A.	66 nm	RO	16.0	1.71–4.82	95.6–97.4%	[36]
ZIF-8	0.34 nm	150 nm	RO	15.5	2.76–3.95	98.9–99.2%	[37]
ZIF-8	0.34 nm	80 nm	RO	15.0	1.11–2.30	98.4–99.4%	[38]

**Table 3:** Modification techniques of membrane and its applications.

S.No.	Modification Techniques of Membrane	Applications
1	Surface coatings	Deposition of layer on membrane surface by physical adsorption process [3]
2	Blending	Modify the bulk morphology by blending of two or more organic and inorganic compounds [43,44]
3	Surface grafting	Addition of functional groups, by plasma treatment as polymerization of mixture of two different gases [45] or by UV irradiation method where free radicals generated upon irradiation by photoinitiated graft polymerization [46].

## Data Availability

Not applicable.
